# Global transcriptional downregulation of TREX and nuclear trafficking machinery as pan-senescence phenomena: evidence from human cells and tissues

**DOI:** 10.1038/s12276-020-00490-x

**Published:** 2020-08-28

**Authors:** Sung Young Kim, Eun Jae Yang, Sung Bae Lee, Young-Sam Lee, Kyoung A. Cho, Sang Chul Park

**Affiliations:** 1 Department of Biochemistry, Konkuk University School of Medicine, Seoul, Korea; 2 Department of New Biology, DGIST, Daegu, Korea; 3 Department of Brain & Cognitive Sciences, DGIST, Daegu, Korea; 4 Protein Dynamics-based Proteotoxicity Control Laboratory, Basic Research Lab, DGIST, Daegu, Korea; 5 Well Aging Research Center, Division of Biotechnology, DGIST, Daegu, Korea; 6 The Future Life and Society Research Center, Chonnam National University, Gwangju, Korea

**Keywords:** Cellular signalling networks, Nuclear pore complex, RNA transport

## Abstract

Nucleocytoplasmic trafficking (NCT) of macromolecules is a fundamental process in eukaryotes that requires tight controls to maintain proper cell functions. Downregulation of the classical NCT pathway in senescent cells has been reported. However, whether this is a hallmark that exists across all types of cellular senescence remains unknown, and whether the mRNA export machinery is altered during senescence has not been demonstrated. Here, we show that the global transcriptomic downregulation of both the TREX (transcription-export) machinery and classical NLS-dependent protein transport machinery is a hallmark of varying types of senescence. A gene set-based approach using 25 different studies showed that the TREX-NCT gene set displays distinct common downregulated patterns in senescent cells versus its expression in their nonsenescent counterparts regardless of the senescence type, such as replicative senescence (RS), tumor cell senescence (TCS), oncogene-induced senescence (OIS), stem cell senescence (SCS), progeria and endothelial cell senescence (ECS). Similar patterns of TREX-NCT gene downregulation were also shown in two large human tissue genomic databases, the Genotype-Tissue Expression (GTEx) and The Cancer Genome Atlas (TCGA) databases. We also found that early-stage cancer tissues show consistent age-related patterns of TREX-NCT enrichment, suggesting the potential significance of TREX-NCT genes in determining cell fate in the early stage of tumorigenesis. Moreover, human cancer tissues exhibit an opposite TREX-NCT enrichment pattern with aging, indicating that deviation from age-related changes in TREX-NCT genes may provide a novel but critical clue for the age-dependent pathogenesis of cancer and increase in cancer incidence with aging.

## Aging: Slowing down of nucleocytoplasmic trafficking

Proteins that move genetic information out of the nucleus and into the rest of the cell may be important in aging, and serve as markers of early-stage cancer. DNA is stored in the cell’s nucleus, and the messages which it encodes must be exported from the nucleus for gene expression. Aging is thought to be linked to a decrease in this export, but the exact mechanism remains unclear. Sung Young Kim, Konkuk University School of Medicine, Seoul, South Korea, and co-workers investigated key nuclear export proteins in healthy, cancerous, and aging cells. They found that nuclear export is strongly decreased in aging cells and shows distinctive patterns in very-early-stage cancer cells. These results shed further light on the cellular basis of aging, and may provide novel biomarkers for early cancer detection.

## Introduction

Cellular senescence is thought to be a fundamental mechanism of aging and tumor suppression characterized by permanent termination of cell proliferation, which may be induced by multiple mechanisms. A variety of cell-intrinsic and cell-extrinsic stresses from replication, chemicals, and oncogenes can induce a cellular senescence program. Immortal cells, such as cancer cells and stem cells, are no exceptions to the senescence process. Furthermore, genetically based accelerated premature senescence models have been established, such as Hutchinson-Gilford progeria syndrome (HGPS) and Werner’s syndrome (WS), both of which are now believed to result from respective single spontaneous gene mutations (lamin A genes in HGPS and WRN genes in WS). Despite varying modes of cellular senescence induction, the fundamental and universal mechanism for the aging process remains elusive.

Nucleocytoplasmic trafficking (NCT) of macromolecules, such as signaling proteins and messenger ribonucleoproteins (mRNPs), is one of the most crucial processes in eukaryotes and requires highly sophisticated control. Classical nuclear trafficking of most proteins and RNAs, except mRNA, depends on Kaps (also known as importin/karyopherin-β-type transport receptors) and the Ran cycle. In contrast, mRNA export is independent of the Kaps and the Ran gradient and depends on a separate export mechanism in which the TREX (transcription-export) complex plays a vital role. The TREX complex, conserved in eukaryotes, is composed of the core THO complex and proteins related to the export and metabolism of mRNA. NXF1 and NXT1, also known as TREX-associated factors (TREX-AFs), are cellular mRNA transporter proteins that cooperate with TREX to facilitate nuclear export. Recently, extensive investigation has been performed on another complex called transcription and export complex 2 (TREX-2), which is suggested to interact with NXF1 and to shuttle between transcription sites and NPC^1^. All these components, TREX, TREX-AFs, and TREX-2 (which we refer to as ‘TREXes’ in this paper), play a crucial role in mRNA export and ‘gene gating’, which links active gene transcription with the export of mRNA (see refs. ^2,3^ for a recent review).

Decreased expression of NCT genes has been reported in a number of senescent cells, which has led to propose a nuclear barrier hypothesis of aging as a fundamental mechanism of aging, mainly based on vast reduction of NCT genes and proteins but without special attention to mRNA trafficking^4–6^. Although significant advances in the understanding mRNA export have been achieved, it has never been determined whether the mRNA export machinery also decreases in senescent cells at the transcriptional similar to the cases of NCT genes. Therefore, in this study, we attempted to clarify this issue by using gene sets from 25 different cellular senescence studies on replicative senescence (RS), tumor cell senescence (TCS), oncogene-induced senescence (OIS), stem cell senescence (SCS), progeria-specific and general endothelial senescence (ES). In addition, we analyzed two massive human tissue transcriptome databases, GTEx and TCGA, to compare and confirm the data in vivo. In further analysis, we paid particular attention to the role of cellular senescence in tumorigenesis.

## Materials and methods

### Data composition

All data used in this paper are publicly available. Seventeen publicly available study data sets (GSE19018, GSE36640, GSE48761, GSE3860, GSE24487, GSE58721, GSE69296, GSE17546, GSE45729, GSE2487, GSE75207, GSE60652, GSE54402, GSE19864, GSE33710, GSE35957, and GSE48662) included only samples that were selected for in vitro senescent cell lines showing five different types of senescence (RS, TCS, OIS, SCS, and progeria) and four types of array platforms (Supplementary Table S2). From the eight publicly available endothelial cell-related genomic studies (GSE45541, GSE37091, GSE77239, GSE13712, GSE54095, E-MEXP-2283, E-MTAB-1388, and E-MTAB-6521), only the samples of senescent endothelial cells and younger counterparts were included (Supplementary Table S3). We excluded animal studies and studies with extremely small sample sizes or inadequate control conditions. Two large RNA-seq (Illumina HiSeq) databases, GTEx and TCGA, were obtained from UCSC Xena Public Data Hub (http://xena.ucsc.edu/). GEO data from Affymetrix platforms were preprocessed using the robust multiarray average (RMA) function, which is implemented in the R affy package. The RMA function includes quantile normalization with background correction, log_2_ transformation, and summarization. Missing values were omitted from the data. Detailed information on the databases (TCGA and GTEx) and the comprehensive procedures for sample collection, RNA extraction, normalization processes, and quality control can be found in previously published papers^7,8^.

### Gene set analysis

Gene set scores for the NCTFs and TREXes were calculated by averaging the normalized gene expression values (z-transformed mean values) per experiment. These scores were used to represent curated subsets of genes with specific expression patterns. The *p*-value, odds ratio (OR), and 95% confidence interval (CI) were calculated using one-sided Fisher exact test for a 2 × 2 table (2-dimensional scatter plot in this paper, with Q1 representing the upper right-hand quadrant; Q2, upper left-hand quadrant; Q3, lower left-hand quadrant; and Q4, right-hand quadrant; for example, if the targeted sample frequency of the gene set in Q3 were significantly smaller than the background frequency or the expected frequency based on random chance, then we would regard the sample as being under-enriched in Q3). Throughout this paper, the focus of the enrichment patterns and odds ratios was on senescence. We used the fisher.test function, which is implemented in R with the following parameters: one-sided, alpha = 0.05, and a null hypothesis stating that the odds ratio equals 1).

### Cell culture

Normal neonatal HDFs (PCS-201-010, American Type Culture Collection) were cultured at Dulbecco’s modified Eagle’s medium supplemented with 10% fetal bovine serum (WELGENE) and 1× antibiotic and antimycotic solution (WELGENE) at 37 °C in 5% CO_2_. The cells were successively passaged, and proliferating (young) and senescent (old) cells were collected after doubling ≤1 day and ≥14 days, respectively. To confirm the senescence phenotype of the old HDFs, senescence-associated β-galactosidase staining was performed with a senescence ß-galactosidase staining kit (Cell Signaling Technology) according to the manufacturer’s instructions.

### Quantitative reverse transcription-polymerase chain reaction (qRT-PCR)

Total RNA was extracted using QIAzol reagent (Qiagen), and 1 μg of RNA was subjected to complementary DNA (cDNA) synthesis using a Transcriptor First Strand cDNA synthesis kit (Roche). qPCR was conducted with 1 μM primer pair for each target gene (Supplementary Table S4) in a 10 μl final volume reaction mixture containing 1× KAPA SYBR Fast qPCR Master Mix (Merck) with a LightCycler 480 Instrument II system (Roche). The relative expression of target genes was estimated using the comparative critical cycle method with RPS11 as a housekeeping control.

All statistical analyses were performed using R version 3.2.3 (R Foundation for Statistical Computing Platform).

## Results

### Aging and downregulation of the TREX-NCTF genes: evidence from human cell models

For dimensionality reduction purposes and a better understanding of the expression change of functional units, we allocated the mRNA export components into three categories: TREX (16 genes), TREX-2 (5 genes), and TREX-associated factors (8 genes), which we collectively refer to as ‘TREXes’ in this paper. Similarly, we generated three main groups of the canonical core NLS-dependent nuclear trafficking machines: nuclear pore complexes (NPCs, 31 genes), nuclear transport receptors (NTRs, 11 genes) and the Ran cycle system (4 genes), which we refer to as ‘NCTFs’. We also curated and further subcategorized NPC into the NPC Y complex, NPC FG (phenylalanine–glycine repeat), NPC TM (transmembrane), and NPC basket based on its functional and structural units. Additionally, we separately categorized the extremely long-lived nucleoporins as a NPC subclass, ELLPs-Nups, because they were recently reported to have half-lives of more than 6 months^9^. Supplementary Table S1 has detailed information on categorized genes, which were recently reviewed in refs. ^2,10–12^.

**Fig. 1:**
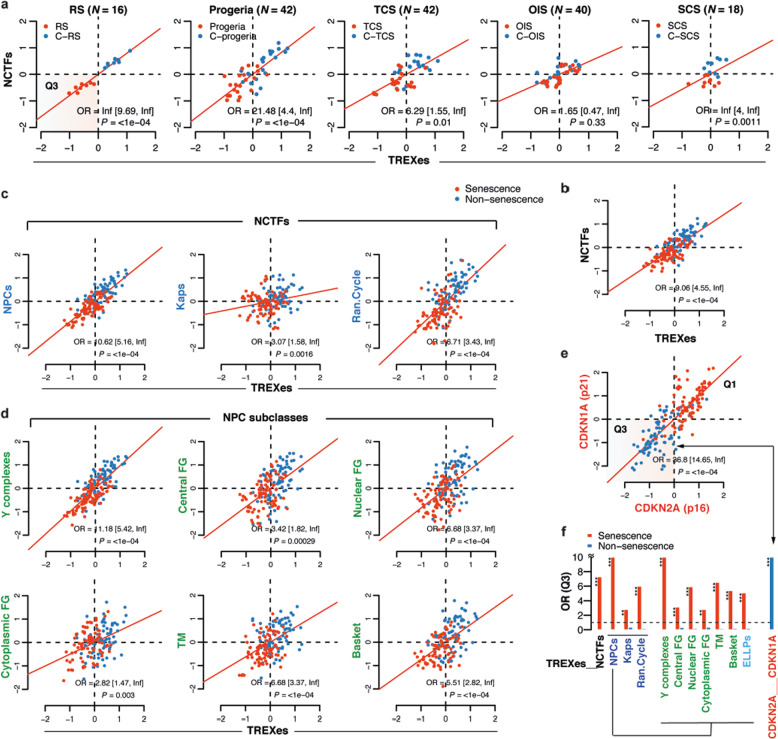
TREXes and NCTFs show distinctive enrichment patterns in various types of senescent cells. **a** Analysis of the two-dimensional differential mean expression of TREXes and NCTFs in five senescent cell types (red dots) of different origins and their young counterparts (blue dots). After gene set mean expressions were z-standardized, the odds ratio (OR) and statistical significance were calculated by Fisher 2-tailed exact test. Horizontal and vertical dashed lines are median points for TREXes and NCTFs. Senescent cells were more likely to be in the lower left-hand quadrant (Q3), and the odds ratios are shown with 95% confidence intervals and *p*-values. **b**–**d** Panel **b** through **d** are compilations of the data on five senescence types showing the enrichment patterns of TREXes and NCTFs. **b** TREXes and NCTFs in all five types of senescent cells. **c** TREXes and subclasses of NCTFs: NPCs, Kaps, and Ran cycle. **d** TREXes and six distinct subclasses of NPC: Y complexes, central FG, nuclear FG, cytoplasmic FG, TM, and basket. **e** Two-dimensional differential mean expression of *CDKN2A* (p16) and *CDKN1A* (p21), well-known senescence markers, in all senescent cell types. **f** Bar plot representing the odds ratios (Q3) of the NCTFs, their subclasses, and TREXes as shown in (**b**) through (**e**). The red bar indicates the odds ratio (Q3) of the senescent cells, and the blue bar indicates the odds ratio of the nonsenescent cells. The color legends on the *x*-axis, i.e., black, blue, green, and red, represent (**b**–**e**), respectively. RS replicative senescence, TCS tumor cell senescence, OIS oncogene-induced senescence, SCS stem cell senescence, Kaps karyopherins, FG phenylalanine–glycine repeat, TM transmembrane.

To efficiently detect subtle expression changes among the genes in functional units displaying high multicollinearity (Supplementary Fig. S1) and to reduce variance, the mean expression levels of each categorized gene were used as variates for the downstream analyses. For convenience, in this paper, we refer to each category (i.e., TREXes, NCTFs and their subclasses) by the mean expression of each categorized gene set. Using seventeen cell-based in vitro studies categorized by senescence phenotype (Supplementary Table S2), we first explored the expression levels of TREXes and NCTFs in senescent cells. Figure 1a shows the extent of TREX and NCTF expression in five distinct types of senescent cells (RS, progeria, TCS, OIS, and SCS) and their nonsenescent counterparts. The senescent cells (red dots) were more likely to be in Q3 compared to the nonsenescent cells (blue dots), which were more prominent in Q1. This finding suggests the downregulation of TREXes and NCTFs in senescent cells compared to their counterparts, which we discovered to be a common feature in various types of senescent cells regardless of the cause for the senescence, such as cancer, stem cells, and cells carrying a single-gene mutation. Figure 1b shows the expression levels of all five senescence models presented in Fig. 1a, dichotomizing senescent and nonsenescent cells. Senescent cells exhibited significant under-enrichment of TREXes and NCTFs in Q1 and over-enrichment in Q3 (OR = 9.06; 95% CI 4.55-Inf; *P* < 1e−04). When we applied the subclasses of the NCTFs separately, a clear enrichment pattern was observed for all three subclasses, which was most pronounced for the NPCs (Fig. 1c, f). The same enrichment patterns were obtained when we applied the subclasses of the TREXes separately (Supplementary Fig. S2).

We then further explored the enrichment pattern of the NPC subclasses, and we observed consistent results regardless of the NPC subgroup, with the most significance in the Y complexes, which are NPC scaffolds (Fig. 1d). Even in the extremely long-lived nucleoporins (the ELLPs-Nups subclass), a clear pattern was observed (Fig. 1f). In contrast, an over-enrichment of senescent cells in Q1 (red dots) was shown when we applied the mean expression of well-known senescence markers *CDKN2A* (which encodes p16 protein) and *CDKN1A* (which encodes p21 protein) (Fig. 1e, f).

**Fig. 2:**
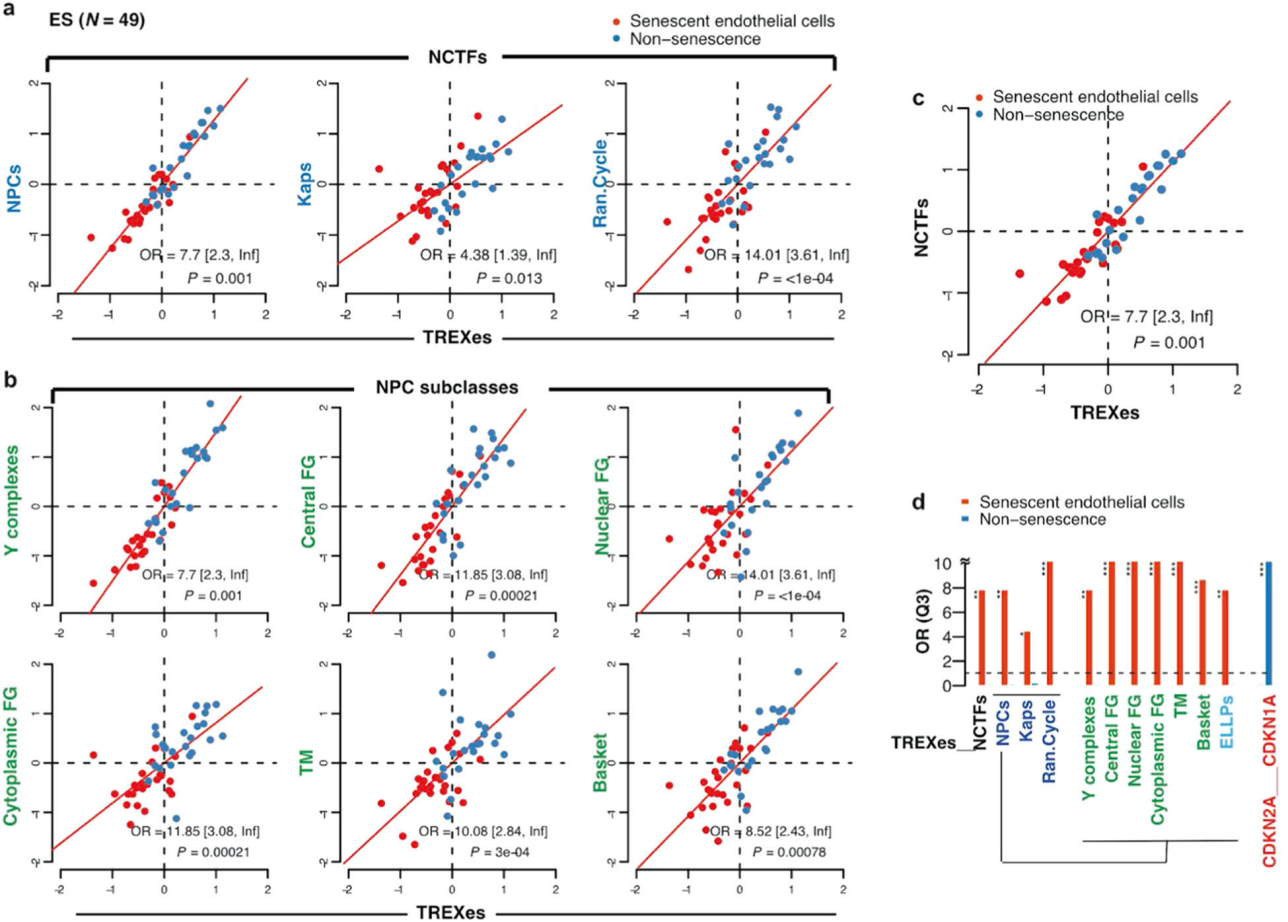
TREXes and NCTFs show distinctive enrichment patterns in endothelial senescent cells. Analysis of two-dimensional differential mean expression of TREXes and NCTFs between endothelial senescent cells (red dots) from 8 screened endothelial aging studies and their young counterparts (blue dots). After gene set mean expression levels were z-standardized, the odds ratio (OR) and statistical significance were calculated by Fisher 2-tailed exact test. Horizontal and vertical dashed lines are median points for the TREXes and NCTFs. Endothelial senescent cells were more likely to be in the lower left-hand quadrant (Q3), and the odds ratios are shown with 95% confidence intervals and *p*-values. **a** TREXes and subclasses of NCTFs: NPCs, Kaps, and Ran cycle genes. **b** TREXes and six distinct subclasses of NPC: Y complexes, central FG, nuclear FG, cytoplasmic FG, TM, and basket. **c** Collective TREXes and NCTFs of endothelial senescent cells. **d** Bar plot representing the odds ratios (Q3) of NCTFs and subclasses and TREXes, as shown in (**a**) through (**c**). The red bar indicates the odds ratio (Q3) of the senescent cells, and the blue bar indicates the odds ratio of the nonsenescent cells. ES endothelial senescence, Kaps karyopherin, FG phenylalanine–glycine repeat, TM transmembrane.

In addition to the senescence of fibroblasts described above, we extended our study to the senescence of endothelial cells to determine their mechanisms in the general mechanism of aging. Endothelial cell senescence is not only an important contributor to vascular aging but also a substantial cause of age-related cardiovascular diseases such as atherosclerotic heart disease, hypertension, and stroke^13,14^. Despite its significance, the underlying mechanism of endothelial cell senescence remains for further research. The expression of TREXes and NCTFs was investigated in a total of 8 in vitro endothelial senescence-associated genomic studies that were screened and categorized based on senescent phenotypes (Supplementary Table S3). The same methods were incorporated for the investigation, and the results displayed consistent enrichment patterns the TREXes and NCTF subclasses in Q3, suggesting their importance in understanding age-related vascular diseases (Fig. 2).

**Fig. 3:**
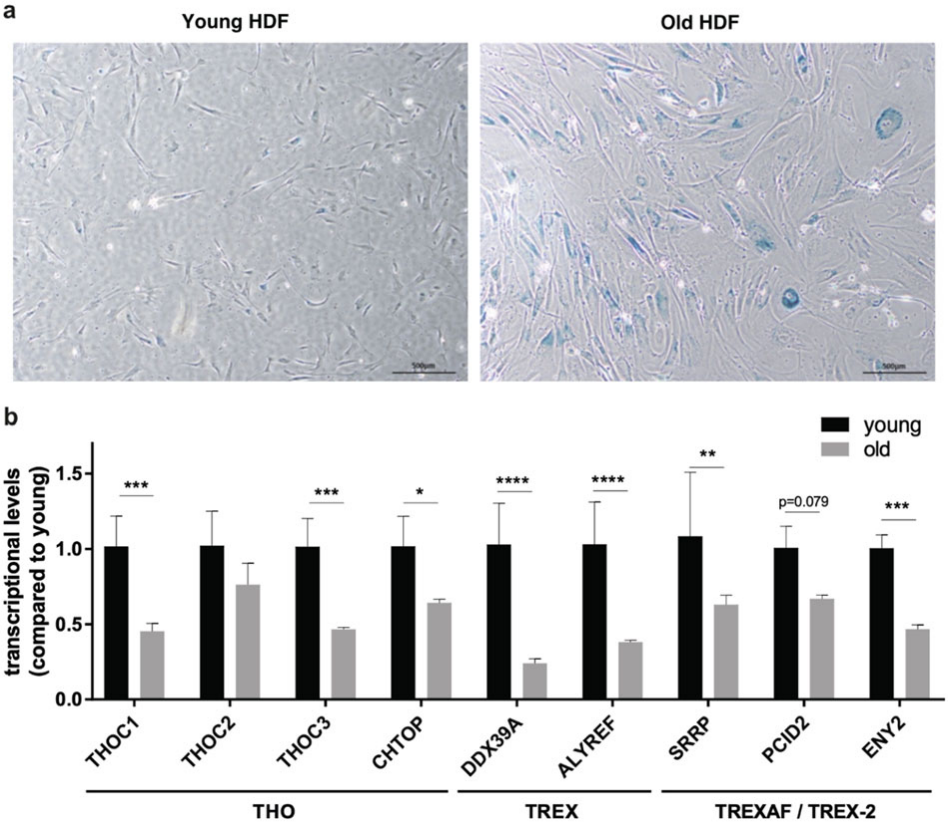
The downregulation of TREXes is associated with cellular senescence. **a** Positive staining of β-galactosidase in replicative senescence models of human diploid fibroblasts (HDFs). Scale bar, 500 μm. **b** qRT-PCR analysis of representative TREXes in the young and old HDFs.

Next, we performed real-time RT-PCR analysis on a subset of TREXes in a replicative cellular senescence model of human diploid fibroblasts. The results validated the overall downregulation observed for the senescent (old) cells, compared with proliferating (young) cells, implying significant alterations to the mRNA export machinery during senescence (Fig. 3).

### Aging and downregulation of the TREX and NCTF genes: evidence from human tissues

**Fig. 4:**
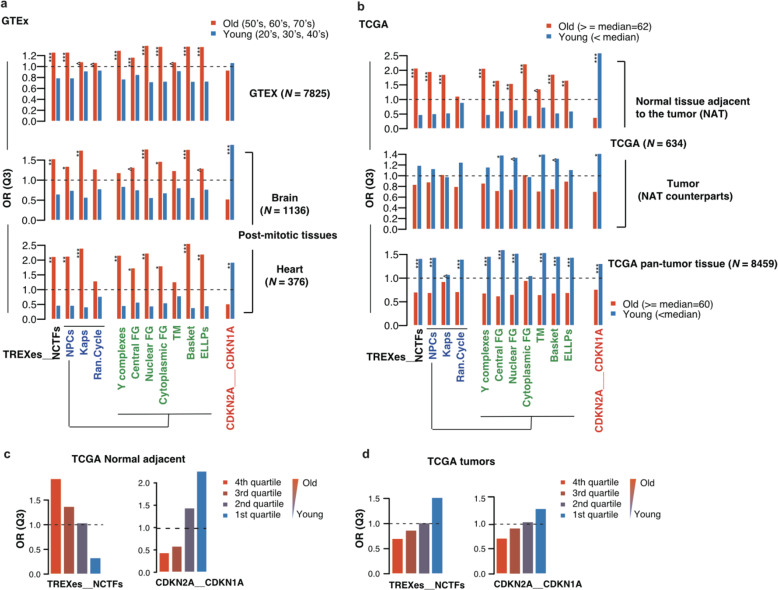
TREXes and NCTFs show distinctive enrichment patterns in both normal human and cancer during aging. **a** Comparison of the odds ratios of the TREXes and NCTF subclasses and *CDKN2A* and *CDKN1A* in the old and young samples from GTEx. Bar plot representing the odds ratios (ORs, Q3) of the NCTFs or their subclasses and TREXes. The red bar indicates these ORs in old tissue and the blue bar in young tissue. Color legends for the *x*-axis are the same as those shown in Fig. 1e. Higher ORs indicate a greater likelihood of data being in Q3 (refer to Fig. 1a), signifying under-enrichment. The top graph represents the results from the total number of samples from the GTEx database. Middle and bottom panel show results from the brain and heart tissues, which are postmitotic tissues. **b** Comparison of the ORs of TREXes and NCTF subclasses and *CDKN2A* and *CDKN1A* in the old and young samples from the TCGA database. The top and middle panels show the results from normal adjacent tissues and their tumor counterparts, respectively. The bottom panel shows the results of the total tumor samples. **c**, **d** Findings shown in (**b**) based on age quartiles.

We next assessed whether the same enrichment patterns were shown in tissues of human origin procured from the GTEx and TCGA databases. GTEx is a comprehensive public resource and contains numerous human samples from ‘normal’, non-diseased tissues. TCGA, one of the most esteemed cancer genomics programs created to date, contains thousands of patient samples in which a portion also include normal adjacent counterparts. We first dichotomized the GTEx sample population based on age, with those in their 50’s, 60’s, and 70’s categorized into the old group and those in their 20’s, 30’s, and 40’s into the young group, because the public GTEx portal provides sample ages in 10-year intervals. Analyzed the same way as in Fig. 1, the old group illustrated in Fig. 4a shows the same enrichment patterns of TREXes and NCTF subclasses with the senescent cells illustrated in Fig. 1, although the odds ratios are not as drastic (Fig. 4a, upper panel and Supplementary Fig. S3a). Interestingly, *CDKN2A* and *CDKN1A* showed only subtle, nonsignificant differences in the odds ratio of the old and young groups in Q3. These results imply that global transcriptional downregulation of TREXes and NCTFs and their subclasses are strongly correlated with aging in normal human tissues as well. Since senescence is also observed in postmitotic tissues and because the expression of the NPC components is associated with mitotic activity^15^, we further examined the modes of TREX and NCTF expression in postmitotic tissues using the GTEx database. We found that these findings are still apparent with even more dramatic evidence in postmitotic tissues, including brain and heart (Fig. 4a, middle and bottom panel).

### Alterations to TREX and NCTF expression in human tumor tissues

In addition, we examined TCGA cancer samples and their normal adjacent counterparts (*N* = 634) and dichotomized the findings based on the median age of the sample population (62 years). As expected, normal adjacent samples from TCGA showed strong over-enrichment patterns in Q3 for the TREXes and NCTFs based on aging (i.e., the increased OR in Q3 indicates the decreased expression of TREXes and NCTFs in the old group), but to our surprise, an opposite enrichment pattern was exhibited with aging in the tumor counterparts (Fig. 4b, upper and middle panel, and Supplementary Fig. S3b). This phenomenon was more prominent when all the tumor tissues, regardless of the normal counterparts (pan-tumoral tissues) were applied in our analysis (Fig. 4b, bottom panel). Interestingly, with regard to the well-known senescence markers, *CDKN2A* and *CDKN1A*, the results were consistent with the findings of the normal tissue, as shown in Fig. 4a; however, the enrichment pattern of *CDKN2A* and *CDKN1A* showed no difference in either normal or tumor tissues. To determine whether the same pattern exists when samples were further subdivided, we categorized the TCGA data into four quartiles by age. We obtained the same results, showing that, in normal adjacent tissues, the ORs for the TREXes and NCTFs decreased with the younger age quartile, while the ORs for *CDKN2A* and *CDKN1A* increased with the younger age (Fig. 4c). In tumor tissues, the ORs of both TREXes and NCTFs and *CDKN2A* and *CDKN1A* increased with the younger age quartile (Fig. 4d).

### Downregulation of TREX and NCT machinery in early-stage cancers

**Fig. 5:**
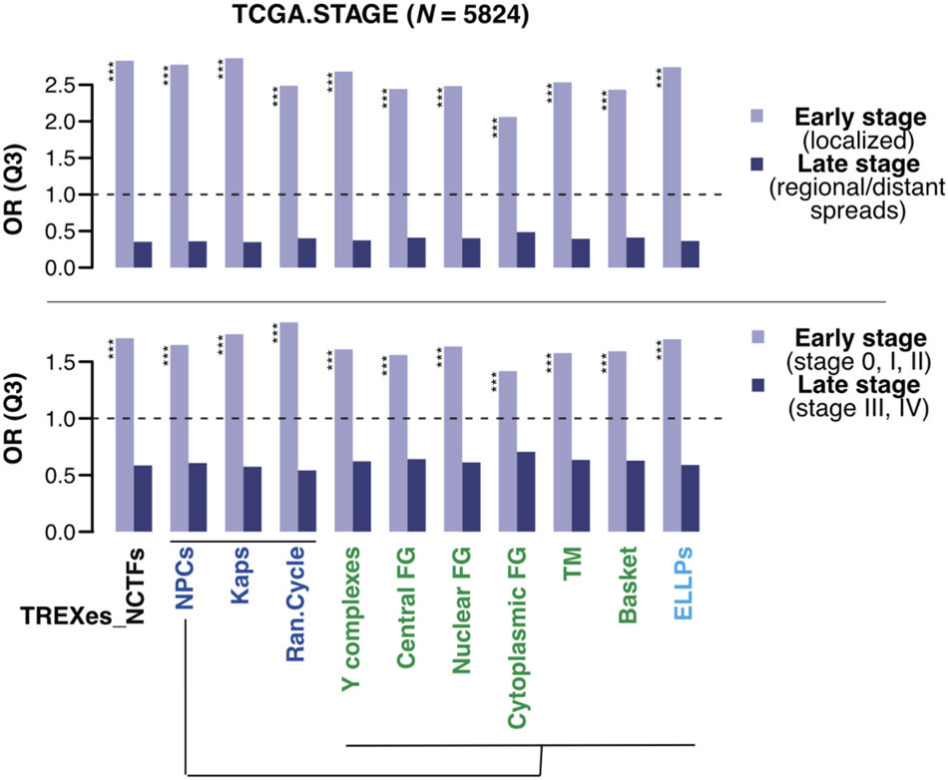
TREXes and NCTFs show distinctive enrichment patterns by cancer stage. Comparison of the odds ratios of TREXes and NCTF subclasses in early- and late-stage cancers, as obtained from the GTEx database. Bar plot representing the odds ratios (ORs, Q3) of the NCTFs or their subclasses and TREXes. The light violet bar indicates the ORs in the early-stage cancer tissue, and the dark violet bar indicates thee ORs in the late-stage cancer tissue. Color legends on the *x*-axis are the same as those shown in Fig. 1e. Higher ORs indicate a greater likelihood of data being in Q3.

Because senescent cells in tumors are detectable only in the early preinvasive stages of tumor progression^16,17^, the enrichment patterns of the TREXes and NCTFs were investigated with regard to tumor stage. Data on the primary tumors with pathologic stage annotation from the normalized pan-cancer TCGA data set were extracted. Because senescent cells are detected solely in noninvasive stages, we dichotomized the samples based on tumor stage. Tumors in stage I, which is the only noninvasive state (i.e., localized cancers), were annotated as ‘early-stage cancers’, and tumors in stages II, III, and IV were annotated as ‘late-stage cancers’ (i.e., regional or distant spreading). Furthermore, we employed alternative labeling of the early- and late-stage cancers by assigning primary tumors in stage I or II to the early-stage group and primary tumors in stage III or IV to the late (advanced) stage group. The same experimental procedure was followed and the prominent over-enrichment patterns of TREXes and NCTF subclasses in Q3 were observed distinctly from the early-stage cancer, which display high similarity to the patterns observed from the aged cells (Fig. 5). This result implies significant roles of TREXes and NCTF in early-stage cancer development, suggesting their potential usage as factors for discriminating early stages of cancer.

## Discussion

NCT of signal proteins and mRNA is a critical step for proper cell function, as almost all cellular activities are linked through this process. Although genes involved in NCT are thought to be of a static nature, maintaining a consistent rate of expression, NCT itself has very dynamic characteristics, as an ordinary mammalian cell has 3000–4000 NPCs, with every NPC facilitating approximately 10^3^ translocations in one second^18^. mRNA trafficking, which is an essential step for gene expression, is subject to rigorous quality control to ensure properly processed mRNA exits the nucleus^12^. Small disruptions to this intricate process, therefore, is thought to have a tremendous impact on downstream pathways, including the cell cycle and cell fate decisions. In this regard, alterations of the nuclear trafficking of proteins and mRNAs likely influence the cellular process of senescence, directly resulting in various senescence-associated phenotypes. Based on these results, the nuclear barrier hypothesis of aging was proposed, which implicates aging-dependent reduction in NCT of both proteins and mRNAs^5,6^, which may result in senescence-related changes in cell morphology and function. The causality between the functional decline of trafficking and senescence has yet to be determined.

Intriguingly, in contrast to the consistent expression patterns of genes that encode p16 and p21 found in both tumor tissues and normal adjacent tissues with aging, a reversed pattern was observed for TREXes and NCTFs, showing the greater disparity between normal and tumor tissues with aging (Fig. 4c, d). The underlying mechanism for this phenomenon is not yet clear, but it is obviously important to explore. A master regulator(s) for TREXes and NCTFs may very likely exist if the genes in these complexes are found to act in unison. Impairment of a master regulator(s), possibly due to the accumulation of mutations with aging, may result in more favorable environments for active RNA export and nuclear trafficking of signaling molecules, which are fundamental processes for cancer cell proliferation. Another proposal suggests that regulator(s) for TREXes and NCTFs have dual roles as oncogenes in tumor cells and as tumor suppressors in normal cells. The TREX-NCTF expression profiles in cancer tissues from older patients suggest their possible functions as oncogenes, not tumor suppressors, in normal tissues. A substantial number of transcription factors and kinases are reported to be both oncogenic and tumor suppressive, and paradoxically, the transcriptional activity of tumor suppressors has been shown to increase in various cancer cells^19–23^. With respect to the cellular senescence mechanism thought to be a fundamental tumor-suppressing process, there appears to be some programmed decline of nuclear trafficking related to aging, and TREXes and NCTF regulator(s) may behave as tumor suppressors with aging. However, in environments prone to cancer, these regulator(s) may exhibit oncogenicity and accelerate cancer development. Deviations from age-related changes in TREXes and NCTFs in cancer tissues may be critical clues to the mechanism of the age-dependent increase in cancer susceptibility. Further studies and rigorous evaluation of the results are urgently needed to determine the key regulatory process(es) of the TREX-NCTF machinery, which may provide new insights into carcinogenesis and novel targets for cancer therapeutics.

The prominent alteration patterns in the preinvasive tumor stage in relation to the expression of TREX-NCTF genes in senescent cells and tissues strongly suggest the potential use of TREXes and NCTFs as useful biomarkers for the detection of early-stage cancers, which may lead to the elucidation of the underlying mechanism. The idea of using these genes as novel tumor-detecting biomarkers is very valuable since early detection and treatment of preinvasive cancer will greatly enhance the survival rate of patients and their overall prognosis.

The accumulation of senescent cells in aged tissues has been indicated, although the actual portion of senescent cells is generally very difficult to measure because of the absence of true universal markers. Currently, most senescence markers are not incapable of rendering false positive signals, including the well-known senescence-associated β-galactosidase. Moreover, markers from cell cultures may not be consistent with those from tissue samples^24^. Therefore, universal senescent markers remain to be discovered. Here, we report a small but clearly significant, decrease in TREXes and NCTFs not only through a variety of aging models but also based on pan-tumor tissue data on aged humans from the GTEx and TCGA databases. Although the levels of senescent cells present in vivo might be very small and specific for each type of tissue, these cells may be critical for the aging-dependent decrease that we found in the TREXes and NCTFs. One of the interesting findings in our study is that postmitotic tissues have similar enrichment patterns.

Many questions remain to be solved to advance our understanding of the aging-dependent reduction in TREX-NCTF expression. Although we have conjectured that the Sp1 transcriptional factor might play an important role in the maintenance of NCT genes^25^, more thorough queries into the ultimate control system for aging-dependent changes in both NCTFs and TREXes are urgently required: how are TREXes and NCTFs transcriptionally deactivated in the aging process? What gene regulators, such as transcription factors, coactivators/corepressors, or microRNAs; epigenetic and genomic alterations, such as chromatin remodeling and copy number variation; and/or other mediators are required for this highly coordinated regulation of TREXes and NCTFs? How do TREXes and NCTFs impact the actions of the various downstream effector pathways and the characteristics of the resulting senescent phenotypes? Further studies on the details of the transcriptional activation and inactivation of TREXes and NCTFs are critical for understanding the fundamental mechanism of senescence progression.

Taken together, the results from gene set analysis clearly demonstrated that global transcriptional downregulation of TREXes and the nuclear trafficking machinery are common to a variety of cellular senescent types in normal human tissues of aging and cancer. These data indicate that the transcriptional downregulation of TREX and NCT genes may be the pan-senescence phenomena, further suggesting that it is the essential nature of the aging process.

## Supplementary information

Supplementary Materials

## References

[1] Umlauf D (2013). The human TREX-2 complex is stably associated with the nuclear pore basket. J. Cell Sci..

[2] Heath CG, Viphakone N, Wilson SA (2016). The role of TREX in gene expression and disease. Biochem. J..

[3] Schubert T, Köhler A (2016). Mediator and TREX-2: emerging links between transcription initiation and mRNA export. Nucleus.

[4] Park SC (2017). Survive or thrive: tradeoff strategy for cellular senescence. Exp. Mol. Med..

[5] Park SC (2011). Nuclear barrier hypothesis of aging as mechanism for trade-off growth to survival. Adv. Exp. Med. Biol..

[6] Kim SY (2010). Senescence-related functional nuclear barrier by down-regulation of nucleo-cytoplasmic trafficking gene expression. Biochem. Biophys. Res. Commun..

[7] GTEx Consortium. (2013). The genotype-tissue expression (GTEx) project. Nat. Genet..

[8] Cancer Genome Atlas Research Network. (2013). The Cancer Genome Atlas Pan-Cancer analysis project. Nat. Genet..

[9] Savas JN, Toyama BH, Xu T, Yates JR, Hetzer MW (2012). Extremely long-lived nuclear pore proteins in the rat brain. Science.

[10] Kabachinski G, Schwartz TU (2015). The nuclear pore complex—structure and function at a glance. J. cell Sci..

[11] Stawicki S, Steffen J (2017). The nuclear pore complex: a comprehensive review of structure and function. Int. J. Acad. Med..

[12] Katahira J (2012). mRNA export and the TREX complex. Biochim. Biophys. Acta.

[13] Donato AJ, Morgan RG, Walker AE, Lesniewski LA (2015). Cellular and molecular biology of aging endothelial cells. J. Mol. Cell. Cardiol..

[14] Ungvari Z, Tarantini S, Donato AJ, Galvan V, Csiszar A (2018). Mechanisms of vascular aging. Circ. Res..

[15] Hetzer MW (2010). The role of the nuclear pore complex in aging of post-mitotic cells. Aging (Albany NY).

[16] Collado M, Serrano M (2006). The power and the promise of oncogene-induced senescence markers. Nat. Rev. Cancer.

[17] Collado M (2005). Tumour biology: senescence in premalignant tumours. Nature.

[18] Ribbeck K, Görlich D (2001). Kinetic analysis of translocation through nuclear pore complexes. EMBO J..

[19] Chae SW (2011). Overexpressions of Cyclin B1, cdc2, p16 and p53 in human breast cancer: the clinicopathologic correlations and prognostic implications. Yonsei Med. J..

[20] Wilson C (2014). The paracrine hormone for the GUCY2C tumor suppressor, guanylin, is universally lost in colorectal cancer. Cancer Epidemiol. Biomark. Prev..

[21] Al Aameri RFH (2017). Tonic suppression of PCAT29 by the IL-6 signaling pathway in prostate cancer: reversal by resveratrol. PLoS ONE.

[22] Ohuchida K (2006). S100A11, a putative tumor suppressor gene, is overexpressed in pancreatic carcinogenesis. Clin. Cancer Res..

[23] Shen L, Shi Q, Wang W (2018). Double agents: genes with both oncogenic and tumor-suppressor functions. Oncogenesis.

[24] Childs BG, Durik M, Baker DJ, van Deursen JM (2015). Cellular senescence in aging and age-related disease: from mechanisms to therapy. Nat. Med..

[25] Kim SY, Kang HT, Han JA, Park SC (2012). The transcription factor Sp1 is responsible for aging-dependent altered nucleocytoplasmic trafficking. Aging Cell.

