# Supplementary material for: Global transcriptional downregulation of TREX and nuclear trafficking machinery as pan-senescence phenomena: evidence from human cells and tissues
Source: Exp Mol Med. 2020 Aug 28;52(8):1351–9. doi: 10.1038/s12276-020-00490-x (PMC8080647; doi:10.1038/s12276-020-00490-x)
Supplement: Supplementary file 1 — Supplementary Materials [file 12276_2020_490_MOESM1_ESM.docx]

**Supplementary Materials for**

**Global transcriptional down-regulation of TREX and nuclear trafficking machinery as pan-senescence phenomena: evidence from human cells and tissues**

**This PDF file includes:**

Supplementary Tables S1 to S4

Supplementary Figures S1 to S3

**Supplementary Tables**

**Supplementary Table S1: Curated list of NCTFs and TREXes genes and related information**

| No. | HGNC | ALIAS | EntrezID | GeneName | SubClass | sub-subclasses |
| --- | --- | --- | --- | --- | --- | --- |
| 1 | *NUP98 | ADIR2 \| NUP196 \| NUP96 \| NUP98 | 4928 | nucleoporin 96kDa and nucleoporin 98kDa | NPC | *NUP98 subcomplex \| Central FG Nups (NUP98) \| ELLPs (NUP98) \| Core scaffold Nups (NUP96) \| Y‐complexes (NUP96) \| Cytoplasmic complexes (NUP98) \| OR (NUP96) |
| 2 | NUP62 | IBSN \| SNDI \| p62 \| NUP62 | 23636 | nucleoporin 62kDa | NPC | Central FG Nups \| NUP62 complex |
| 3 | NUP54 | NUP54 | 53371 | nucleoporin 54kDa | NPC | Central FG Nups \| NUP62 complex |
| 4 | NUP58 | NUPL1 \| PRO2463 \| NUP58 \| NUP45 | 9818 | nucleoporin 58kDa | NPC | Central FG Nups \| NUP62 complex |
| 5 | NUP88 | NUP88 | 4927 | nucleoporin 88kDa | NPC | *NUP214 subcomplex \| Linker Nups \| Cytoplasmic complexes |
| 6 | NUP210 | GP210 \| POM210 \| NUP210 | 23225 | nucleoporin 210kDa | NPC | TM |
| 7 | POM121 | P145 \| POM121A \| POM121 | 9883 | POM121 transmembrane nucleoporin | NPC | FG Nups \| TM |
| 8 | NDC1 | NET3 \| TMEM48 \| NDC1 | 55706 | NDC1 transmembrane nucleoporin | NPC | TM |
| 9 | NUP153 | HNUP153 \| N153 \| NUP153 | 9972 | nucleoporin 153kDa | NPC | Nuclear FG Nups \| Basket complexes |
| 10 | NUP50 | NPAP60 \| NPAP60L \| NUP50 | 10762 | nucleoporin 50kDa | NPC | Nuclear FG Nups \| Basket complexes |
| 11 | TPR | TPR | 7175 | translocated promoter region, nuclear basket protein | NPC | \| Basket complexes |
| 12 | NUP205 | C7orf14 \| NUP205 | 23165 | nucleoporin 205kDa | NPC | ELLPs \| Core scaffold Nups \| IR |
| 13 | NUP188 | KIAA0169 \| hNup188 \| NUP188 | 23511 | nucleoporin 188kDa | NPC | ELLPs \| Core scaffold Nups \| IR |
| 14 | NUP155 | ATFB15 \| N155 \| NUP155 | 9631 | nucleoporin 155kDa | NPC | ELLPs \| Core scaffold Nups \| IR |
| 15 | NUP93 | NIC96 \| NUP93 | 9688 | nucleoporin 93kDa | NPC | ELLPs \| Linker Nups \| Core scaffold Nups \| IR |
| 16 | NUP35 | MP-44 \| MP44 \| NP44 \| NUP53 \| NUP35 | 129401 | nucleoporin 35kDa | NPC | Core scaffold Nups \| IR |
| 17 | NUP107 | NUP84 \| NUP107 | 57122 | nucleoporin 107kDa | NPC | ELLPs \| Core scaffold Nups \| Y‐complexes \| OR |
| 18 | NUP160 | NUP160 | 23279 | nucleoporin 160kDa | NPC | ELLPs \| Core scaffold Nups \| Y‐complexes \| OR |
| 19 | NUP43 | bA350J20.1 \| p42 \| NUP43 | 348995 | nucleoporin 43kDa | NPC | ELLPs \| Core scaffold Nups \| Y‐complexes \| OR |
| 20 | NUP37 | p37 \| NUP37 | 79023 | nucleoporin 37kDa | NPC | Core scaffold Nups \| Y‐complexes \| OR |
| 21 | AHCTF1 | ELYS \| MST108 \| MSTP108 \| TMBS62 \| AHCTF1 | 25909 | AT-hook containing transcription factor 1 | NPC | Core scaffold Nups \| Y‐complexes \| OR |
| 22 | NUP133 | hNUP133 \| NUP133 | 55746 | nucleoporin 133kDa | NPC | Core scaffold Nups \| Y‐complexes \| OR |
| 23 | NUP85 | FROUNT \| Nup75 \| NUP85 | 79902 | nucleoporin 85kDa | NPC | Core scaffold Nups \| Y‐complexes \| OR |
| 24 | SEC13 | D3S1231E \| SEC13L1 \| SEC13R \| npp-20 \| SEC13 | 6396 | SEC13 homolog, nuclear pore and COPII coat complex component | NPC | Core scaffold Nups \| Y‐complexes \| OR |
| 25 | SEH1L | SEC13L \| SEH1A \| SEH1B \| Seh1 \| SEH1L | 81929 | SEH1-like nucleoporin | NPC | Core scaffold Nups \| Y‐complexes \| OR |
| 26 | AAAS* | AAA \| AAASb \| ADRACALA \| ADRACALIN \| ALADIN \| GL003 \| AAAS | 8086 | achalasia, adrenocortical insufficiency, alacrimia | NPC | NUP214 subcomplex \| OR |
| 27 | NUP214 | CAIN \| CAN \| D9S46E \| N214 \| p250 \| NUP214 | 8021 | nucleoporin 214kDa | NPC | NUP214 subcomplex \| Cytoplasmic FG \|Cytoplasmic complexes |
| 28 | RANBP2 | ADANE \| ANE1 \| IIAE3 \| NUP358 \| TRP1 \| TRP2 \| RANBP2 | 5903 | RAN binding protein 2 | NPC | NUP214 subcomplex \| Cytoplasmic FG \|Cytoplasmic complexes |
| 29 | NUPL2 | CG1 \| NLP-1 \| NLP_1 \| hCG1 \| NUPL2 | 11097 | nucleoporin like 2 | NPC | NUP214 subcomplex \| Cytoplasmic FG \|Cytoplasmic complexes |
| 30 | GLE1 | GLE1L \| LCCS \| LCCS1 \| hGLE1 \| GLE1 | 2733 | GLE1 RNA export mediator | NPC | NUP214 subcomplex \| Cytoplasmic complexes, NPC ­associated |
| 31 | RAE1 | MIG14 \| MRNP41 \| Mnrp41 \| dJ481F12.3 \| dJ800J21.1 \| RAE1 | 8480 | ribonucleic acid export 1 | NPC | NUP98 suncomplex \| Cytoplasmic complexes, NPC ­associated |
| 32 | KPNA1 | IPOA5 \| NPI-1 \| RCH2 \| SRP1 \| KPNA1 | 3836 | karyopherin alpha 1 (importin alpha 5) | NTF |  |
| 33 | KPNA2 | IPOA1 \| QIP2 \| RCH1 \| SRP1alpha \| KPNA2 | 3838 | karyopherin alpha 2 (RAG cohort 1, importin alpha 1) | NTF |  |
| 34 | KPNA3 | IPOA4 \| SRP1 \| SRP1gamma \| SRP4 \| hSRP1 \| KPNA3 | 3839 | karyopherin alpha 3 (importin alpha 4) | NTF |  |
| 35 | KPNA4 | IPOA3 \| QIP1 \| SRP3 \| KPNA4 | 3840 | karyopherin alpha 4 (importin alpha 3) | NTF |  |
| 36 | KPNA5 | IPOA6 \| SRP6 \| KPNA5 | 3841 | karyopherin alpha 5 (importin alpha 6) | NTF |  |
| 37 | KPNA6 | IPOA7 \| KPNA7 \| KPNA6 | 23633 | karyopherin alpha 6 (importin alpha 7) | NTF |  |
| 38 | KPNB1 | IMB1 \| IPO1 \| IPOB \| Impnb \| NTF97 \| KPNB1 | 3837 | karyopherin (importin) beta 1 | NTF |  |
| 39 | XPO1 | CRM1 \| emb \| exp1 \| XPO1 | 7514 | exportin 1 | NTF |  |
| 40 | NUTF2 | NTF2 \| PP15 \| NUTF2 | 10204 | nuclear transport factor 2 | NTF |  |
| 41 | TNPO1 | IPO2 \| KPNB2 \| MIP \| MIP1 \| TRN \| TNPO1 | 3842 | transportin 1 | NTF |  |
| 42 | TNPO2 | IPO3 \| KPNB2B \| TRN2 \| TNPO2 | 30000 | transportin 2 | NTF |  |
| 43 | RAN | ARA24 \| Gsp1 \| TC4 \| RAN | 5901 | RAN, member RAS oncogene family | RAN.CYCLE |  |
| 44 | RANBP1 | HTF9A \| RANBP1 | 5902 | RAN binding protein 1 | RAN.CYCLE |  |
| 45 | RANGAP1 | Fug1 \| RANGAP \| SD \| RANGAP1 | 5905 | Ran GTPase activating protein 1 | RAN.CYCLE |  |
| 46 | RCC1 | CHC1 \| RCC1-I \| SNHG3-RCC1 \| RCC1 | 1104 | regulator of chromosome condensation 1 | RAN.CYCLE |  |
| 47 | THOC1 | HPR1 \| P84 \| P84N5 \| THOC1 | 9984 | THO complex 1 | THO |  |
| 48 | THOC2 | CXorf3 \| MRX12 \| MRX35 \| THO2 \| dJ506G2.1 \| hTREX120 \| THOC2 | 57187 | THO complex 2 | THO |  |
| 49 | THOC3 | THO3 \| hTREX45 \| THOC3 | 84321 | THO complex 3 | THO |  |
| 50 | THOC5 | C22orf19 \| Fmip \| PK1.3 \| fSAP79 \| THOC5 | 8563 | THO complex 5 | THO |  |
| 51 | THOC6 | BBIS \| WDR58 \| fSAP35 \| THOC6 | 79228 | THO complex 6 | THO |  |
| 52 | THOC7 | NIF3L1BP1 \| fSAP24 \| hTREX30 \| THOC7 | 80145 | THO complex 7 | THO |  |
| 53 | DDX39A | BAT1 \| BAT1L \| DDX39 \| DDXL \| URH49 \| DDX39A | 10212 | DEAD (Asp-Glu-Ala-Asp) box polypeptide 39A | TREX |  |
| 54 | DDX39B | BAT1 \| D6S81E \| UAP56 \| DDX39B | 7919 | DEAD (Asp-Glu-Ala-Asp) box polypeptide 39B | TREX |  |
| 55 | ALYREF | ALY \| ALY/REF \| BEF \| REF \| THOC4 \| ALYREF | 10189 | Aly/REF export factor | TREX |  |
| 56 | FYTTD1 | UIF \| FYTTD1 | 84248 | forty-two-three domain containing 1 | TREX |  |
| 57 | LUZP4 | CT-28 \| CT-8 \| CT28 \| HOM-TES-85 \| LUZP4 | 51213 | leucine zipper protein 4 | TREX |  |
| 58 | CHTOP | C1orf77 \| FL-SRAG \| FOP \| SRAG \| SRAG-3 \| SRAG-5 \| pp7704 \| CHTOP | 26097 | chromatin target of PRMT1 | TREX |  |
| 59 | SARNP | CIP29 \| HCC1 \| HSPC316 \| THO1 \| SARNP | 84324 | SAP domain containing ribonucleoprotein | TREX |  |
| 60 | POLDIP3 | PDIP46 \| SKAR \| POLDIP3 | 84271 | polymerase (DNA-directed), delta interacting protein 3 | TREX |  |
| 61 | ZC3H11A | ZC3HDC11A \| ZC3H11A | 9877 | zinc finger CCCH-type containing 11A | TREX |  |
| 62 | ERH | DROER \| ERH | 2079 | enhancer of rudimentary homolog (Drosophila) | TREX |  |
| 63 | NXF1 | MEX67 \| TAP \| NXF1 | 10482 | nuclear RNA export factor 1 | TREXAF \| NTF |  |
| 64 | NXT1 | MTR2 \| P15 \| NXT1 | 29107 | nuclear transport factor 2-like export factor 1 | TREXAF \| NTF |  |
| 65 | ZC3H18 | NHN1 \| ZC3H18 | 124245 | zinc finger CCCH-type containing 18 | TREXAF |  |
| 66 | SRRT | ARS2 \| ASR2 \| serrate \| SRRT | 51593 | serrate, RNA effector molecule | TREXAF |  |
| 67 | NCBP1 | CBP80 \| NCBP \| Sto1 \| NCBP1 | 4686 | nuclear cap binding protein subunit 1, 80kDa | TREXAF |  |
| 68 | NCBP3 | C17orf85 \| ELG \| HSA277841 \| NCBP3 | 55421 | nuclear cap binding subunit 3 | TREXAF |  |
| 69 | XPO5 | exp5 \| XPO5 | 57510 | exportin 5 | TREXAF |  |
| 70 | DDX19B | DBP5 \| RNAh \| DDX19 | 11269 | DEAD-box helicase 19B | TREXAF |  |
| 71 | MCM3AP | GANP \| "germinal-centre associated nuclear protein" \| KIAA0572 \| Map80 \| SAC3 | 8888 | minichromosome maintenance complex component 3 associated protein | TREX-2 |  |
| 72 | PCID2 | FLJ11305 | 55795 | PCI domain containing 2 | TREX-2 |  |
| 73 | *SHFM1 (SEM1) | C7orf76 \| "chromosome 7 open reading frame 76" \| SHFD1 \| SHFM1 \| "split hand/foot malformation (ectrodactyly) type 1" | 7979 | SEM1, 26S proteasome complex subunit | TREX-2 |  |
| 7 | ENY2 | "enhancer of yellow 2 homolog (Drosophila)" | 56943 | ENY2, transcription and export complex 2 subunit | TREX-2 |  |
| 75 | CETN2 | Cdc31, Centrin 2 and centrin 3 | 1069 | Cdc31 Also part of centrosome | TREX-2 |  |

| **Table S2. Characteristics of individual studies** | | | | | |
| --- | --- | --- | --- | --- | --- |
| Senescence Types | Dataset | Sample size | | Origin of cell line | Platform |
|  |  | C | S |  |  |
| RS | GSE19018  (Stab BR et al., 2010) | 3 | 3 | IMR90 (PD 30) under 20% oxygen Senescent cells (PD 48) under 20% oxygen | Affymetrix Human Genome U133 Plus 2.0 Array |
|  | GSE36640  (Shah PP et al., 2013) | 5 | 5 | IMR90 (PD 28) and IMR90 (PD 90) | Affymetrix Human Genome U133 Plus 2.0 Array |
| Premature Aging | GSE48761  (Cheung HH et al., 2014) | 10 | 10 | Normal skin fibroblast and WRN mutant fibroblast | Affymetrix Human Gene 1.0 ST Array [transcript (gene) version] |
|  | GSE3860  (Csoka AB et al., 2005) | 3 | 3 | Normal fibroblasts and HGPS fibroblast | Affymetrix Human Genome U133A Array |
|  | GSE24487  ( Liu G et al., 2011) | 2 | 2 | BJ (CRL-2522) fibroblasts and HGPS fibroblast | Affymetrix Human Genome U133 Plus 2.0 Array |
| TCS  (TIS & RIS) | GSE58721  (Zhang G et al., 2016) | 2 | 2 | WM9 melanoma cells  BRAF inhibitor PLX4720 treated TICCS | Illumina HumanHT-12 V4.0 expression beadchip |
|  | GSE69296  (Diep CH et al., 2016) | 6 | 6 | Ovarian ES-2 and senescence cells via FOXO1-dependent induction of p21 and p15 (CDKN2B). | Illumina HumanHT-12 V4.0 expression beadchip |
|  | GSE17546  (Yildiz G et al., 2013) | 6 | 6 | Immortal and senescence-programmed clones from HCC-derived Huh7 cell line. | Affymetrix Human Genome U133 Plus 2.0 Array |
|  | GSE45729  (Lang A et al., 2016) | 2 | 4 | MCF7 breast carcinoma cells were exposed to gamma IR (20 Gy) | Agilent-014850 Whole Human Genome Microarray 4x44K G4112F |
| OIS | GSE2487  (Collado M et al., 2005) | 2 | 4 | IMR90, RAS-induced OIS | Affymetrix Human Genome U133A Array |
|  | GSE75207  (Tordella L et al., 2016) | 3 | 3 | IMR90, RAS-induced OIS | Affymetrix Human Gene 1.0 ST Array [transcript (gene) version] |
|  | GSE60652  (Takebayashi S et al., 2015) | 2 | 2 | IMR90, RAS-induced OIS | Affymetrix Human Genome U133 Plus 2.0 Array |
|  | GSE54402  (Nelson DM et al., 2014) | 5 | 5 | IMR90, RAS-induced OIS | Affymetrix Human Genome U133 Plus 2.0 Array |
|  | GSE19864  (Chicas A et al., 2010) | 4 | 4 | IMR90, RAS-induced OIS | Affymetrix Human Genome U133 Plus 2.0 Array |
|  | GSE33710  (Benhamed M et al ., 2012) | 3 | 3 | WI-38, RAS-induced OIS | Affymetrix Human Genome U133A Array |
| SCS | GSE35957  (Benisch P et al., 2012) | 5 | 5 | hMSC and RS-induced senescent MSC | Affymetrix Human Genome U133 Plus 2.0 Array |
|  | GSE48662  (Sepúlveda JC et al., 2014) | 4 | 4 | hMSC and gamma-IR-induced senescent MSC | Agilent-026652 Whole Human Genome Microarray 4x44K v2 (Probe Name version) |

| **Table S3. Characteristics of individual studies (endothelial cells)** | | | | |
| --- | --- | --- | --- | --- |
| Dataset | Sample size | | Origin of cell line | Platform |
|  | C | S |  |  |
| GSE45541  (Wong PF et al., 2017) | 2 | 3 | Young and replicative senescent of human microvascular endothelial cells-lung (HMVEC-L) | Agilent-014850 Whole Human Genome Microarray 4x44K G4112F (Feature Number version) |
| GSE37091  (Jong HL et al., 2013) | 2 | 3 | Gene signature of young and replicative senescent human umbilical vein endothelial cells (HUVECs) | Agilent-014850 Whole Human Genome Microarray 4x44K G4112F (Probe Name version) |
| GSE77239  (Costarelli L et al., 2017) | 3 | 3 | Young and senescent HCAECs | [HG-U133_Plus_2] Affymetrix Human Genome U133 Plus 2.0 Array |
| GSE13712  (Mun GI et al., 2009) | 3 | 3 | Young and senescent HUVECs | [HG-U133_Plus_2] Affymetrix Human Genome U133 Plus 2.0 Array |
| GSE54095  (Guerrero A et al., 2014) | 4 | 5 | Early and late passage endothelial cells | [HuGene-1_0-st] Affymetrix Human Gene 1.0 ST Array [transcript (gene) version] |
| E-MEXP-2283 (Hofer Edith E et al., 2011) | 2 | 2 | Human umbilical vein endothelial cells during senescence (HUVECs) | Affymetrix GeneChip Human Genome U133 Plus 2.0 [HG-U133_Plus_2] |
| E-MTAB-1388  (Reinhold J et al., 2013) | 4 | 4 | Human umbilical cord blood derived Outgrowth Endothelial Cells (OECs) at early and late passages | Illumina human WG6 BeadChip v3 (HumanWG-6_V3_0_R0_11282955_A) |
| E-MTAB-6521  (An Aerts et al., 2018) | 3 | 3 | Young and senescent HUVECs | Affymetrix GeneChip Human Gene 1.0 ST Array [HuGene-1_0-st-v1] |

**Table S4. List of sequence of qPCR primers**

| **Gene** | **Primer (5’ to 3’)** | |
| --- | --- | --- |
| THOC1 | Forward | AGACAAGGGAACACATGCCC |
|  | Reverse | CTGGTTGGTTGGCTGGAAGA |
| THOC2 | Forward | TCACCCTTCTCCATCACATTCC |
|  | Reverse | TTTCCGCTCTTTCCTGTCCTT |
| THOC3 | Forward | CATCAACGCCCATCCTTCCAA |
|  | Reverse | ACGCCAGCATTTTCCCATCAT |
| DDX39A | Forward | GCCACCCTACAGCAGATTGAG |
|  | Reverse | GACCACCGAAGAACACAGACA |
| ALYREF | Forward | GCCAAAACAACTTCCCGACAA |
|  | Reverse | GCCTTCTTCAGCGTTCCAAAT |
| CHTOP | Forward | TTAAAGCAGCGCCTGGGTAA |
|  | Reverse | GAGCAGGTTTTGACCTCGGA |
| SRRT | Forward | CGCAAAACCAACGACAAGGAT |
|  | Reverse | GGTTCCGCTTCTTGCTACTCT |
| PCID2 | Forward | TCCTCATCCTGGAGAAGCTGA |
|  | Reverse | GCCTTTGACGTGTCCCATGT |
| ENY2 | Forward | AGCCGAAGAGTGTGGTTAGC |
|  | Reverse | CAGTGTGCCTTCAACTGATCC |
| RPS11 | Forward | GAGACTATCTGCACTACATCCGC |
|  | Reverse | AGCGCACTGTCTTGCTCAG |


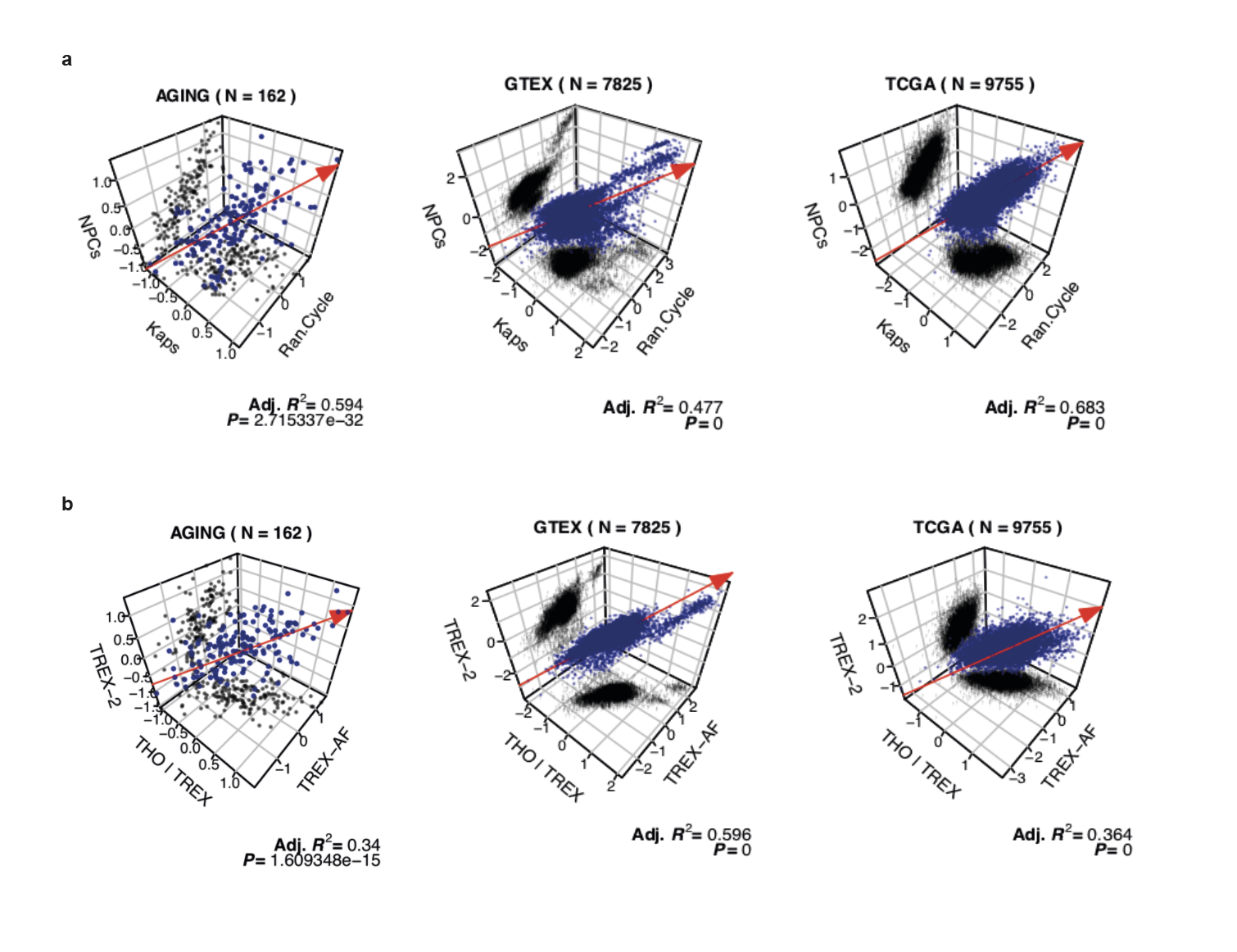


**Supplementary Fig. S1: 3D sample scatter plot for curated gene-set expressions. a** The subgroup of NCTFs have high cross-correlations. Curated gene sets related to NCTFs mainly classified into three groups : genes that code for components of Kaps, Ran cycle and NPC complexes. Average expression of each group specifying the x, y, z coordinates of point. **b** The subgroup of TREXes have high cross-correlations. Curated gene sets related to TREXes mainly classified into three groups : genes that code for components of TREX (THO | TREX), TREX associate factors (TREX-AF) and TREX-2 complexes. The red arrow indicates the regression line.


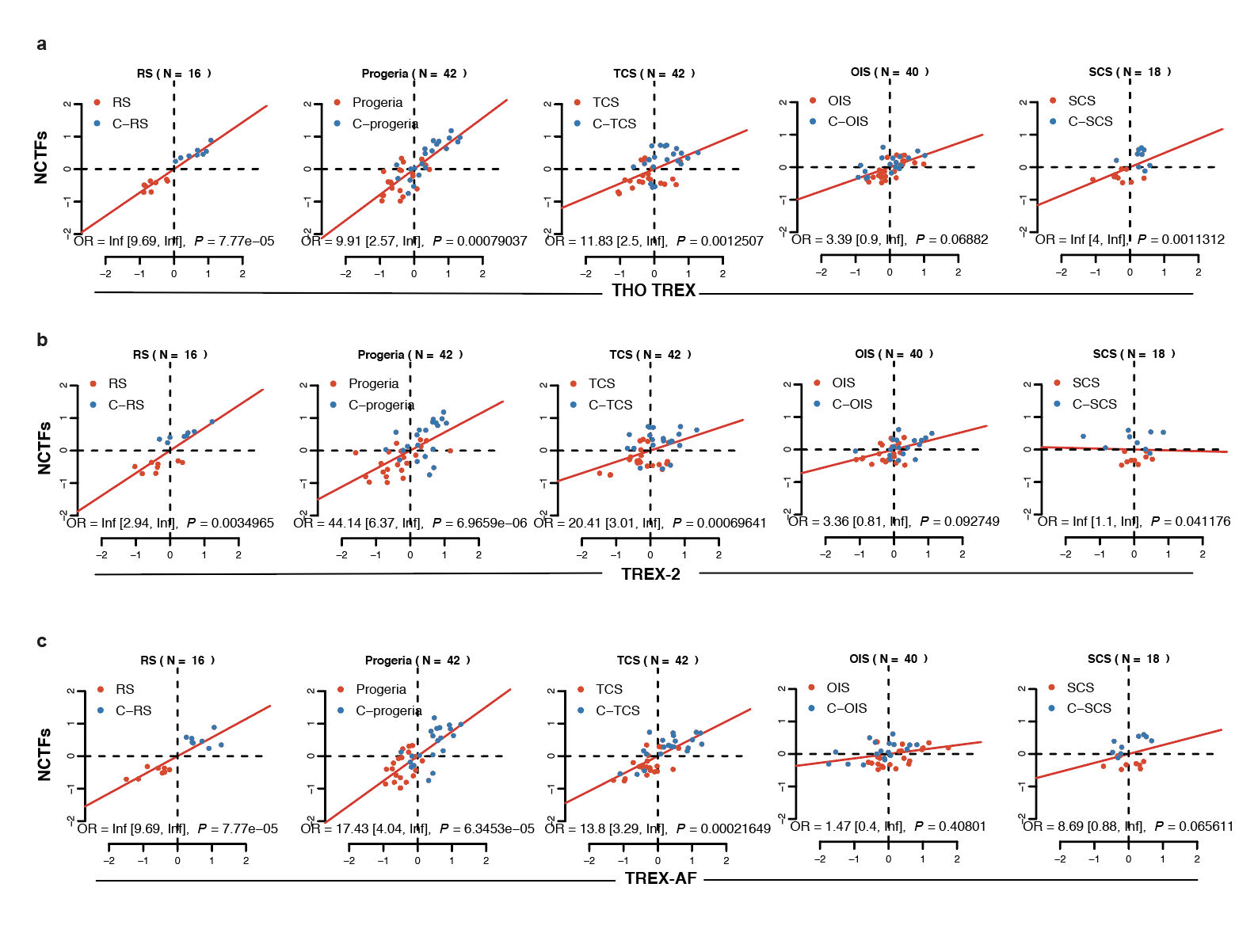


**Supplementary Fig. S2: TREXes subclasses-NCTFs show distinctive enrichment patterns in all types of senescence.** **a, b, c** Analysis of two-dimensional differential mean expression of THO TREX (a), TREX-2 (b), TREX-associated factors (TREX-AF) (c) and NCTFs between five senescent cell types (red dots) of different origin and their young counterparts (blue dots). After gene set mean expressions were z-standardized, odds ratio (OR, Q3) and statistical significance were calculated by Fisher 2-tailed exact test. Horizontal and vertical dashed lines are median points for TREXes subclasses and NCTFs. Senescent cells were more likely to be in lower left-hand quadrant (Q3) and the odds ratios were shown with 95% confidence intervals and p-value. RS, replicative senescence; TIS, treatment induced senescence; OIS, oncogene induced senescence; SCS, stem cell senescence; Kaps, karyopherin; FG, phenylalanine-glycine repeats; TM, transmembrane.


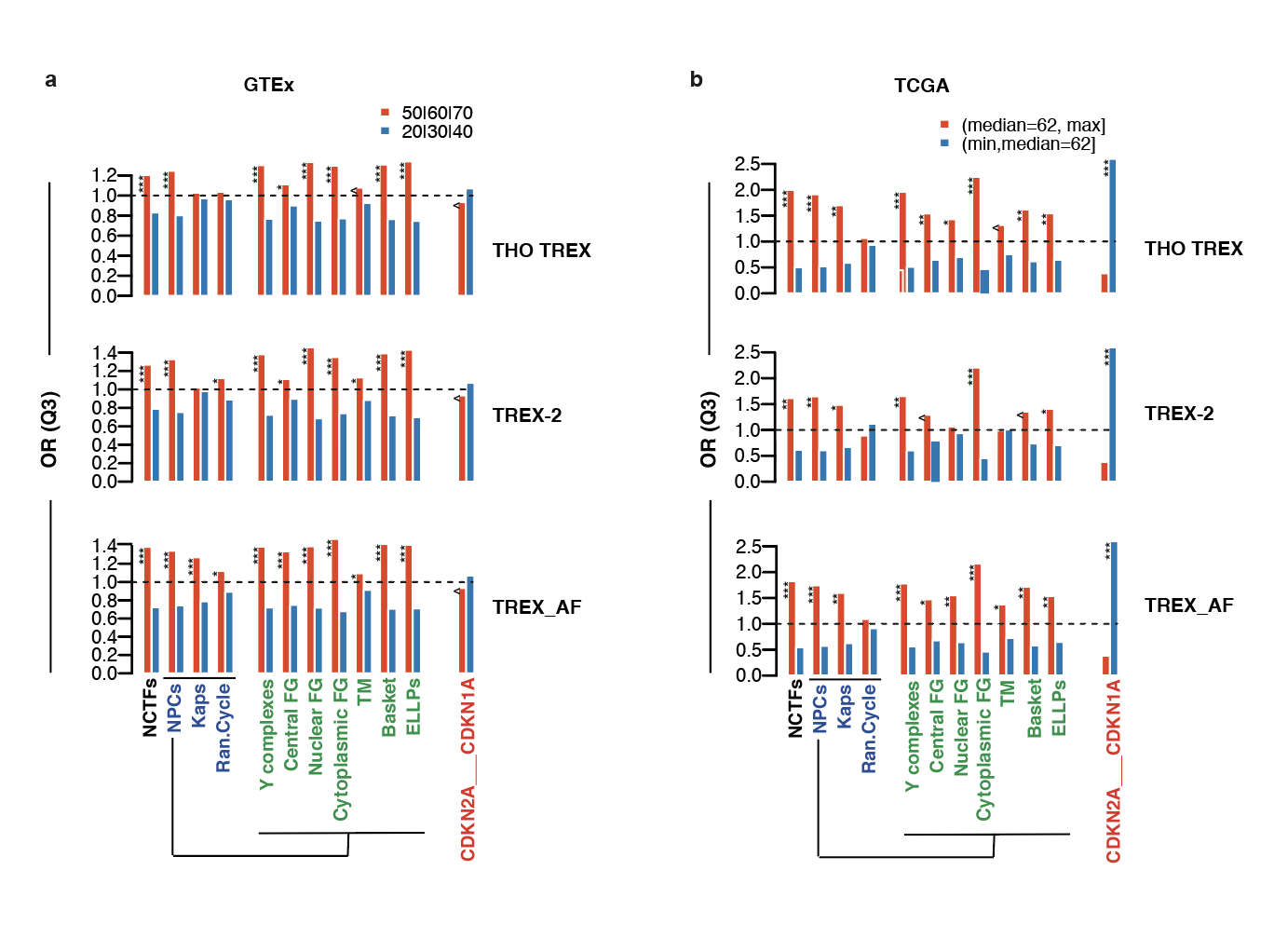


**Supplementary Fig. S3: TREXes subclasses-NCTFs show distinctive enrichment patterns in normal human aging.** **a, b** Comparison of odds ratios (ORs) of TREXes subclasses-NCTF, and *CDKN2A*-*CDKN1A*, in the old and young samples from GTEX (a) and TCGA (b). Bar plot representing the odds ratios (Q3) of NCTFsand TREXes subclasses (upper: THO TREX; middle: TREX-2; bottom; TREX associated factors). Red bar indicates these OR in old tissue and blue bar in young tissue. Refer to Fig. 1e for the color legends on x-axis. Higher ORs indicate the higher likelihood of being found in Q3 (refer to Fig. 1a), signifying under-enrichment.
